# Variations of the hepatobiliary vasculature including coexistence of accessory right hepatic artery with unusually arising double cystic arteries: case report and literature review

**DOI:** 10.1007/s12565-013-0219-5

**Published:** 2013-12-06

**Authors:** Michał Polguj, Michał Podgórski, Piotr Hogendorf, Mirosław Topol

**Affiliations:** 1 Department of Angiology, Medical University of Łódź, Narutowicza Street 60, 90-136 Lodz, Poland; 2 Department of General and Transplant Surgery, Barlicki University Hospital, Medical University of Łódź, Kopcińskiego Street 22, 90-153 Lodz, Poland; 3 Department of Normal and Clinical Anatomy, Medical University of Łódź, Narutowicza Street 60, 90-136 Lodz, Poland

**Keywords:** Hepatobiliary arterial anatomy, Double cystic arteries, Accessory right hepatic artery, Anatomical variation

## Abstract

Familiarity with the different anatomical variations of the arterial supply of the gallbladder and liver is of great importance in all hepatobiliary surgical procedures. A complex anomaly of the hepatobiliary arterial system, which has never been reported before, was found during anatomical dissection of a 73-year-old male Caucasian cadaver. The accessory right hepatic artery (aRHA) took its origin from the gastroduodenal artery. Two cystic arteries were present, the first arising from the gastroduodenal artery (more distal than the aRHA) and the second directly from the aRHA. Potential clinical implications of this anomaly and embryology are discussed. Knowledge of the different anatomical variations of the arterial supply of the gallbladder and liver is of great importance in hepatobiliary surgical procedures.

## Introduction

According to the textbook scheme, the cystic artery is a single vessel that originates from the right branch of the proper hepatic artery (PHA). It usually travels through the hepatobiliary triangle, which is bounded superiorly by the inferior surface of the liver, inferiorly by the cystic duct and medially by the common hepatic duct. In Calot’s triangle, which was initially introduced to describe this region, the superior border is established by the cystic artery (Chen et al. 2000). On approaching the gallbladder, the cystic artery divides into superficial and deep branches that run on the anterior and posterior facets of the gallbladder, respectively. Variations of the origin and course of the cystic artery are very common. Thus, since laparoscopic cholecystectomy became the gold standard for treatment of cholelithiasis, knowledge of anatomical variations of the hepatobiliary arterial system has gained in importance. Blood vessel injuries during laparoscopic cholecystectomy, including cystic artery bleeding, result in conversion to open surgery in up to 1.9 % of cases, causing mortality of about 0.02 % (Ding et al. 2007). Awareness of other possible anomalies in this region is of paramount information for safe cholecystectomy as well as for proper interpretation of liver arteriograms. To our knowledge, the coexistence of accessory right hepatic artery (aRHA) with double cystic arteries described herein has not been previously reported.

## Case report

**Fig. 1:**
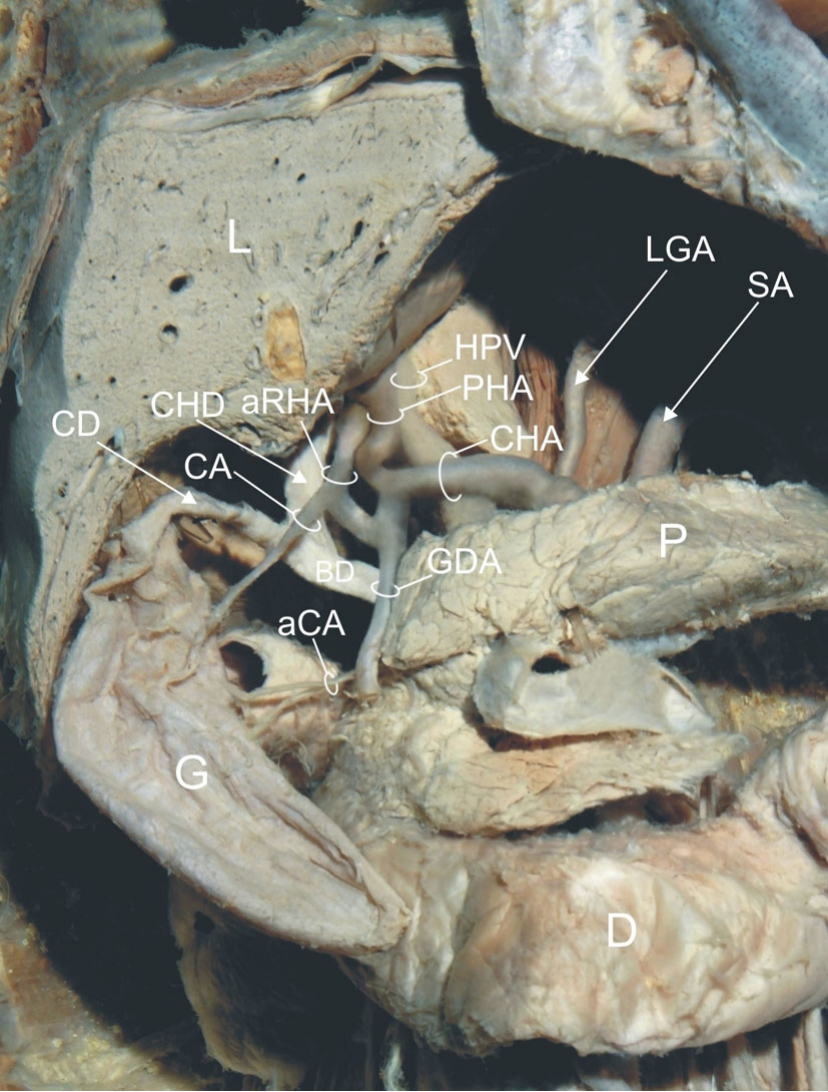
Structures of the hepatobiliary region. *aCA* accessory cystic duct, *aRHA* accessory right hepatic artery, *BD* bile duct, *CA* cystic artery, *CD* cystic duct, *CHA* common hepatic artery, *CHD* common hepatic duct, *D* duodenum, *G* gallbladder, *GDA* gastroduodenal artery, *HPV* hepatic portal vein, *L* liver, *LGA* left gastric artery, *P* pancreas, *PHA* proper hepatic artery, *SA* splenic artery

**Fig. 2:**
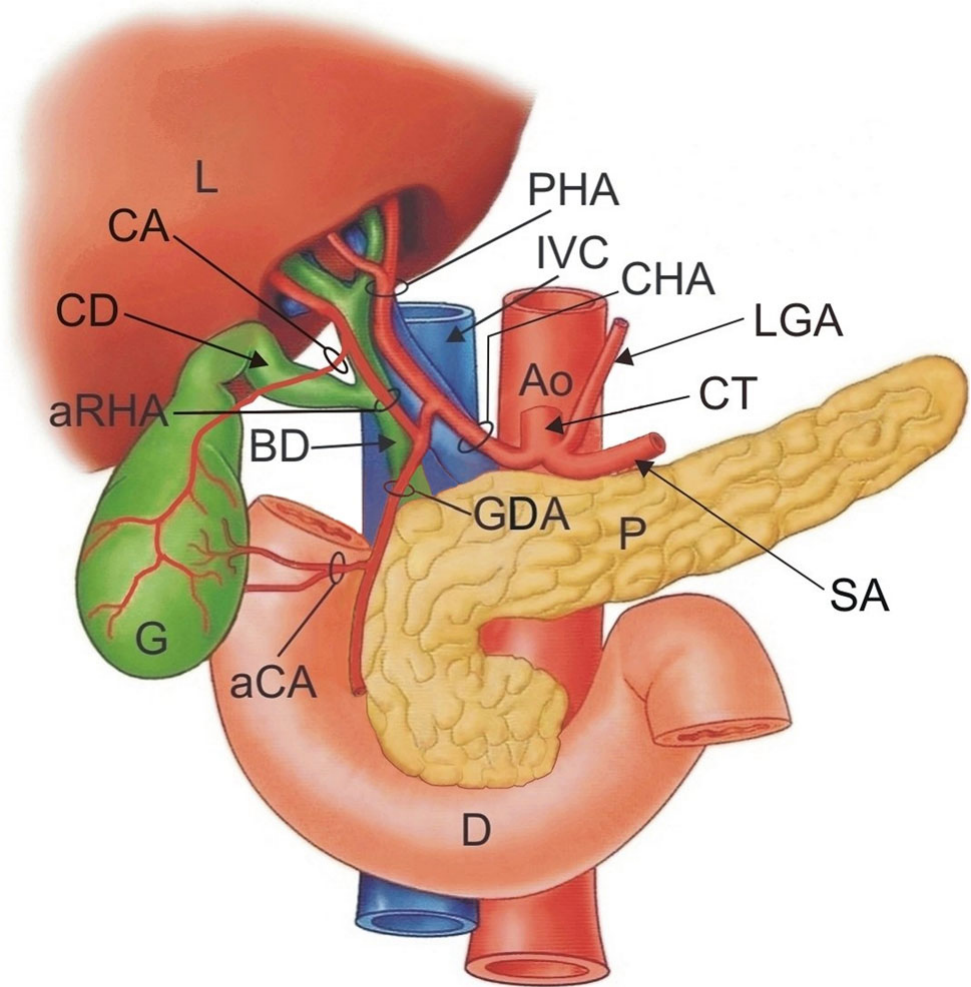
Schematic drawing of the structures of the hepatobiliary region. *Ao* aorta, *aCA* accessory cystic artery, *aRHA* accessory right hepatic artery, *BD* bile duct, *CA* cystic artery, *CD* cystic duct, *CHA* common hepatic artery, *CT* coeliac trunk, *D* duodenum, *G* gallbladder, *GDA* gastroduodenal artery, *IVC* inferior vena cava, *L* liver, *LGA* left gastric artery, *P* pancreas, *PHA* proper hepatic artery, *SA* splenic artery

During anatomical dissection of the abdominal cavity of a 73-year-old male Caucasian cadaver, multiple anatomical variations of the hepatobiliary arterial system were recognised. The common hepatic artery (CHA) took its origin from a coeliac trunk. After giving rise to the gastroduodenal artery (GDA), it travelled to the porta hepatis as the PHA, where it bifurcated into right and left branches. An aRHA with 7.4 mm diameter originated from the GDA, one centimetre below its beginning. The aRHA ran medially and superficially to the common bile duct, entering into the right lobe of the liver (Figs. 1, 2). At the level of the junction of the common hepatic duct with the cystic duct, the aRHA gave rise to the cystic artery (CA). The CA diameter was 5.1 mm. It travelled in front of the bile duct (BD) and then through the hepatobiliary triangle to the region located 23 mm below the neck of the gallbladder. There, it divided into superficial and deep branches.

The gastroduodenal artery travelled inferiorly. Two centimetres distal from the beginning of the aRHA, GDA gave rise to the accessory cystic artery (aCA) (Figs. 1, 2). Its diameter was 2.3 mm. The aCA ascended superolaterally, approaching the middle part of the gallbladder. Afterwards, it bifurcated into superficial and deep branches. The accessory cystic artery did not pass through the hepatobiliary triangle, coursing inferiorly and parallel to the cystic duct without crossing it. No other variations or anomalies were noted in the branching patterns of the hepatobiliary arterial system or in coeliac trunk branches and superior mesenteric artery.

In this case, the blood supply to the liver was supported by three arteries (right and left hepatic arteries—branches of the hepatic artery proper and the aRHA—arising from the gastroduodenal artery), while the gallbladder was supplied by two arteries (the cystic artery arising from the aRHA and accessory cystic artery arising from gastroduodenal artery). The distribution of vessel diameters is presented in Table 1.

**Table 1:** Diameter of investigated arteries

Name of artery	Diameter (mm)
Common hepatic artery (CHA)	14.2
Accessory right hepatic artery (aRHA)	7.4
Proper hepatic artery (PHA)	10.5
Gastroduodenal artery (GDA)	9.8
Cystic artery (CA)	5.1
Accessory cystic artery (aCA)	2.3

## Discussion

The incidence of double cystic artery ranges from 2 to 25 % (Bincy and Somayaji 2010; Ding et al. 2007; Saidi et al. 2007; Suzuki et al. 2000), but its occurrence varies among different populations. It is often associated with congenital absence of the deep branch of the cystic artery (Balija et al. 1999). Double cystic artery can be divided depending on position with respect to the hepatobiliary triangle, bile ducts and portal vein (Balija et al. 1999; Ding et al. 2007; Suzuki et al. 2000). Ding et al. (2007) named cases where the cystic arteries existed not only in the hepatobiliary triangle, but also outside it, i.e. the compound cystic artery type. In their research, only 3 of 600 (0.5 %) Chinese patients had a cystic artery travelling through the hepatobiliary triangle, superficial to the cystic duct, with the accessory artery approaching the gallbladder outside the triangle, as in our case. The same pattern was present in 13 (5.3 %) of 244 Japanese patients operated by Suzuki et al. (2000), who generally reported occurrence of double cystic artery in 27 cases (11.1 %). Zubair et al. (2012) recognised this type in 5.46 % of 220 Pakistani patients who underwent laparoscopic cholecystectomy. Nevertheless, the most common variation, which was seen in 26 (11.8 %) cases, was the double cystic artery passing through the hepatobiliary triangle. On the other hand, in a study carried out in Pakistan (Talpur et al. 2010), double cystic artery was present in only 3 of 300 cases (1 %). Saidi et al. (2007), in 102 Nairobian liver dissections, found double cystic artery in eight cases (7.8 %), and Futara et al. (2001) reported a frequency of 10 % in Ethiopians. In European populations of Slovenians and Croatians, double cystic artery was reported in 13.6 and 5.5 % of cases, respectively (Balija et al. 1999; Mlakar et al. 2003). None of the aforementioned studies included a case of a complex anomaly concerning occurrence of double cystic artery together with aRHA.

Regarding the origins of double cystic arteries, they usually arise from the right hepatic artery or its branches (Mlakar et al. 2003; Suzuki et al. 2000). On the contrary, the most common origins for aberrant cystic arteries include the left, proper or common hepatic arteries, the gastroduodenal artery, the superior pancreaticoduodenal artery and the superior mesenteric artery (Sarkar and Roy 2000). A cystic artery starting from the gastroduodenal artery or from its branches is called a low-lying cystic artery. Such an artery approaches the gallbladder beyond the hepatobiliary triangle (i.e. through the cholecystoduodenal ligament) and is found more superficially in laparoscopy, being at high risk of intersection (Balija et al. 1999). The prevalence of this anatomic variation ranges from 1 to 30 % (Balija et al. 1999; Chen et al. 2000; Sarkar and Roy 2000).

Variations in hepatic arteries are also common. In most variants the aRHA, being the vessel that supports the main artery with greatest lumen, originates from the superior mesenteric artery, PHA or aorta (Ding et al. 2007; Polguj et al. 2010). Only in 1–2 % does it arise from gastroduodenal artery (Futara et al. 2001). The aRHA usually lies deeply under the cystic duct and gallbladder (Ding et al. 2007), but in our case it ran parallel and medial to the common hepatic and bile ducts. Additionally, it travelled closer to the hepatobiliary triangle than the right hepatic artery. Therefore, such an accessory artery becomes the first structure encountered in dissection of the inferior border of the hepatobiliary triangle during laparoscopy, and has a chance of accidental injury. Furthermore, the described variant is a compelling case for transplantation teams, because insufficient blood supply to the liver can lead to graft loss due to tissue hypoxia and parenchymal biliary complications (Koops et al. 2004). Besides, knowledge of exact liver vasculature is vital for interventional radiologists performing chemo- or radio-embolisation of hepatic arteries as well as chemotherapy pump placement (Lewandowski et al. 2007; Sahani et al. 2004).

Embryology is necessary to understand the formation of arterial variations in the liver of a foetus. During foetal development, arterial supply of the liver comes from the CHA, the right hepatic artery originating from the superior mesenteric artery and the left hepatic artery arising from the left gastric artery; thus, complete or partial persistence of the foetal pattern may result in anatomical variations of vascularisation of the liver (Osawa et al. 2004; Polguj et al. 2010). Miyaki’s (1989) investigations corroborated this fact as well; the frequency of aRHA has been stated to be 18.3 % in human foetuses. In adults it was even more common, occurring in an estimated 2.5–5.0 % of cases (Covey et al. 2002; Zahoi et al. 2007). Such observations support the theory of the development of an aRHA by complete or partial persistence of the foetal vascularisation.

All the aforementioned anomalies usually occur separately. Cases of anomalies with double cystic artery associated with variation in hepatic arteries are very rare. Bincy and Somayaji (2010) reported a case of 2 cystic arteries that both arose from PHA just after the former gave rise to the accessory left hepatic artery. Loukas et al. (2006) described double cystic arteries arising from both the right hepatic artery and the posterior superior pancreaticoduodenal artery coexisting with an accessory left hepatic artery arising from a left gastric artery. It is suggested that all these variations can be explained on a developmental basis (Bincy and Somayaji 2010). During development, the liver and gallbladder arise from an intestinal diverticulum (Saidi et al. 2007). The gallbladder is supplied primordially by the coeliac and mesenteric arteries (Loukas et al. 2006). In foetal life, liver receives arterial blood from three sources: the PHA from the CHA, the right hepatic artery from the superior mesenteric artery and the left hepatic artery from the left gastric artery (Douard et al. 2006). As development progresses, vessels regress with a highly variable pattern (Bincy and Somayaji 2010). This explains the high prevalence of anatomical variations in the origins and branching patterns of the hepatobiliary arterial system.

Hugh et al. (1992) described the frequency of initially intersecting the cystic artery in laparoscopic surgery as 6 %. This is particularly pertinent to our case report, where the cystic artery did not pass through the hepatobiliary triangle, and as it had a diameter of 5.1 mm as opposed to 2.3 mm, it may have been the dominant blood supply. This signifies that damage to the artery has greater risk of inducing haemorrhage (Chen et al. 1999; Torres et al. 2009).

Kano et al. (1994) described that injury to the bile duct is the most common major complication of laparoscopic cholecystectomy. This usually stems from mistaken identification of the cystic duct or cystic artery. They also stated that exposure of the cystic duct and cystic artery in the same field of vision is important for preventing such injury (Kano et al. 1994).

In conclusion, haemorrhage and bile leakage are the most common cause for conversion to open surgery during laparoscopic cholecystectomy and usually occur due to variants of structures of the hepatobiliary triangle (Kano et al. 1994; Torres et al. 2009). Preoperative diagnosis of these variants by means of routine investigations is difficult and seen only in exceptional cases (Talpur et al. 2010).

Knowledge of the different anatomical variations of the arterial supply of the gallbladder and liver is of great importance in hepatobiliary surgical procedures. Thus, for safe and uneventful cholecystectomy, especially by means of laparoscopic techniques, it is important to be familiar with anatomic variations in the hepatobiliary arterial system, even if they are very rare, like the one described in this report.
